# Cooperative Downloading for LEO Satellite Networks: A DRL-Based Approach

**DOI:** 10.3390/s22186853

**Published:** 2022-09-10

**Authors:** Hongrok Choi, Sangheon Pack

**Affiliations:** School of Electrical Engineering, Korea University, Seoul 02841, Korea

**Keywords:** deep reinforcement learning (DRL), soft actor-critic (SAC), low earth orbit (LEO) satellite, graph attention network (GAT)

## Abstract

In low earth orbit (LEO) satellite-based applications (e.g., remote sensing and surveillance), it is important to efficiently transmit collected data to ground stations (GS). However, LEO satellites’ high mobility and resultant insufficient time for downloading make this challenging. In this paper, we propose a deep-reinforcement-learning (DRL)-based cooperative downloading scheme, which utilizes inter-satellite communication links (ISLs) to fully utilize satellites’ downloading capabilities. To this end, we formulate a Markov decision problem (MDP) with the objective to maximize the amount of downloaded data. To learn the optimal approach to the formulated problem, we adopt a soft-actor-critic (SAC)-based DRL algorithm in discretized action spaces. Moreover, we design a novel neural network consisting of a graph attention network (GAT) layer to extract latent features from the satellite network and parallel fully connected (FC) layers to control individual satellites of the network. Evaluation results demonstrate that the proposed DRL-based cooperative downloading scheme can enhance the average utilization of contact time by up to 17.8% compared with independent downloading and randomly offloading schemes.

## 1. Introduction

With the global coverage of satellite networks and the development of communication technologies, a wide range of applications, including data collection and remote sensing [1,2,3], surveillance [4], and global broadband Internet access [5], are emerging. In particular, unlike geostationary earth orbit (GEO) satellites and medium earth orbit (MEO) satellites, which are located at altitudes of about 36,000 km and 2000∼36,000 km, respectively, low earth orbit (LEO) satellites are located at altitudes of 500∼2000 km and can guarantee better signal quality and lower propagation delay [6]. Therefore, they are perceived as an attractive platform for edge computing [7,8,9] and a key enabler for ubiquitous 5G and 6G services [10].

In remote sensing and surveillance applications as major applications of LEO satellite networks, when LEO satellites meet ground stations (GSs), they need to transmit collected data to GSs (i.e., download) for further data processing or delivery. Unlike GEO satellites’ relatively stationary mobility from the point of view of a GS, LEO satellites orbit the earth with high mobility. This mobility of LEO satellite imposes the following unique characteristics. First of all, the amount of transmittable data of a LEO satellite highly depends on its contact time with GS, which is determined by its orbit. In addition, the type and the amount of the retaining data of the LEO satellite are highly affected by its trajectory. For example, a satellite that has passed through deserts or suburbs will likely have a smaller amount and less important types of data compared with those that have passed urban areas. In addition, a satellite can collect more important data when it rotates its orbit during the daytime than the night time.

To deliver data efficiently for a given time, there have been studies on data compression for satellite networks [11,12]. However, in these studies, loss of original data is inevitable owing to the compression process, and they require additional resources and time for the compression and decompression processes. Meanwhile, ref. [13,14,15] investigated sum rate maximization problems. Dong et al. [13] considered an integrated terrestrial–satellite network aided by an intelligent reflecting surface (IRS), which is a promising technology for satellite communications in terms of security [16], energy saving [17], and performance improvement [18] by making incoming signals constructive or destructive by shifting phases. They proposed an iterative algorithm with the objective to maximize the weighted sum rate of all users. Khan et al. [14,15] tackled the spectrum scarcity issue in LEO satellites, and they proposed a cognitive-radio (CR)-enabled satellite network using the rate splitting multiple access (RSMA) technology, where GEO and LEO satellites work as primary and secondary nodes, respectively. They also formulated a joint problem of user association and beam resource management for LEO satellites and proposed a greedy algorithm with relaxation techniques. However, they only considered a single LEO satellite; no communications links between LEO satellites were considered.

On the other hand, refs. [19,20,21] investigated data-transmission scheduling problems. Castaing et al. [19] proposed a greedy data -scheduling algorithm for satellite networks consisting of multiple satellites and GSs. Wang et al. [20] formulated an integer linear programming (ILP) problem using a graph considering the resources of satellites and proposed an iterative algorithm for maximizing the sum of successfully scheduled tasks’ priorities. He et al. [21] formulated a joint optimization problem of observation and transmission for agile earth-observing satellite networks and proposed a semi-definite relaxation method and genetic algorithm. However, the above-mentioned studies were unable to resolve the issue of the under-utilization of communication resources when the satellites with sufficient contact time do not have a plenty of data.

Recently, inter-satellite communication-link (ISL)-utilizing cooperative downloading approaches have been also investigated to maximize throughput and solve the resource-under-utilization issue [22,23,24]. They leveraged data offloading through ISLs to distribute an appropriate amount of data to each satellite. Jia et al. [22] constructed a graph reflecting the states of satellite-to-ground communication links (SGLs) and ISLs, and then proposed an iterative algorithm adjusting the download time and the offloading data volume of each satellite. Zhang and Zhou [23] proposed an iterative algorithm considering the energy efficiency of satellites as well as data throughput. He et al. [24] constructed a task flow graph and formulated an ILP problem to maximize the amount of offloaded tasks, which was solved by a genetic algorithm. Even though the use of ISLs is promising for data download services, the previous works assumed relatively stationary environments in that all states are known in advance and unchanged while applying iterative algorithms.

Meanwhile, in modern networks that are increasingly complex and expanding horizontally/vertically, deep-reinforcement-learning (DRL)-based optimization, which can guarantee high performance with low complexity, has received great attention [25]. In particular, its utility has been proven in dynamic networks such as vehicular networks [26] and aerial networks [27]. Along with these successful applications, DRL has also been considered as a promising approach for satellite networks [28,29,30,31]. Wang et al. [28] proposed a DRL-based handover scheme for highly dynamic LEO satellite networks. Tang et al. [29] proposed a resource management scheme based on DRL to guarantee QoS for large-scale satellite-supported remote Internet-of-things (IoT) networks. Huang et al. [30] proposed a DRL-based power allocation scheme for an RSMA-applied 6G LEO system. Yoo et al. [31] investigated a federated learning (FL)-combined DRL system for UAV/LEO satellites to efficiently provide communication resources for ground nodes. However, there has not been a DRL-based cooperative downloading scheme for satellite networks.

In this paper, we try to maximize the amount of the downloaded data of satellite networks where multiple LEO satellites and their ISLs are considered. To this end, we formulate the cooperative downloading problem as a Markov decision problem (MDP) with the objective to maximize the amount of downloaded data to GS. To solve the formulated problem, we adopt a soft-actor-critic (SAC)-based DRL algorithm for discretized action space to learn the dynamics of satellite networks and train the optimal policy. For SAC-based training, we design a novel neural network consisting of (1) a graph attention network (GAT) at the input layer to aggregate graph-oriented network states and (2) parallel fully connected (FC) layers at the output layer to control the individual behaviors of satellites. Evaluation results show that the proposed scheme can enhance the average contact time utilization by up to 17.8% compared with independent downloading and randomly offloading schemes, even when initial data distributions are highly biased.

The contributions of this paper can be summarized as follows: (1) owing to the high dynamics (e.g., mobility, transmission rate, and trajectory) of LEO satellites, it is quite complex to find the optimal policy for cooperative downloading using conventional approaches. Thus, we introduce the use of DRL to solve the problem, which is a promising approach since high-performance servers are planned to be put into orbits [32]. To the best of our knowledge, this is the first DRL work for cooperative downloading in satellite networks; (2) by means of the SAC algorithm for the discretized action space and the neural network design including GAT and parallel FC layers, the proposed DRL-based cooperative downloading framework can effectively learn the dynamics of LEO satellite networks and the optimal policy; and (3) the presented evaluation results provide meaningful insight for future mega-constellations.

The rest of the paper is organized as follows. The system model of LEO satellite networks is described in Section 2. The MDP problem is formulated and the proposed cooperative download scheme is presented in Section 3. The evaluation results and the concluding remarks are given in Section 4 and Section 5, respectively.

## 2. System Model

**Figure 1:**
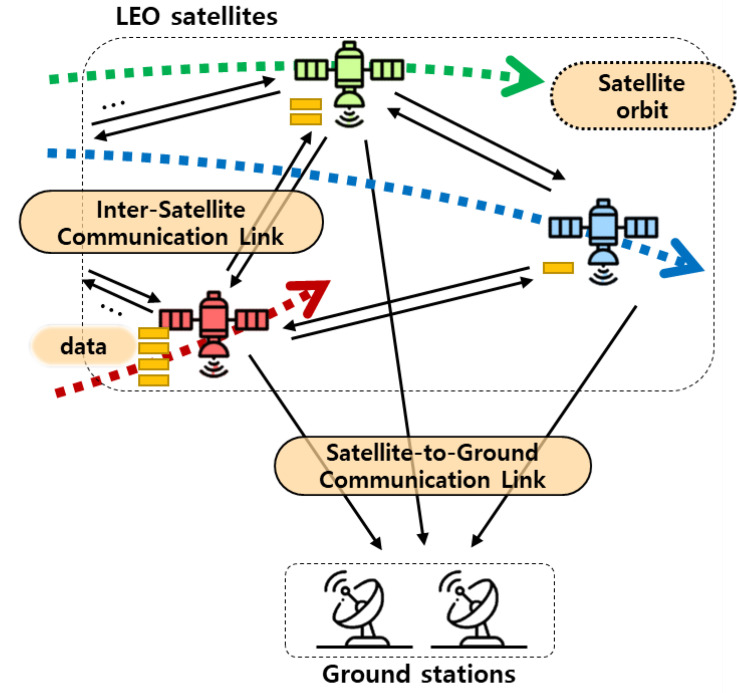
System model.

Figure 1 shows a system model considered in this paper. LEO satellites collect data while traveling along their assigned orbits and contact GSs in a specific area. During the contact time, satellites can download data to GSs through SGLs. Since all satellites have different amounts of data and contact times according to their trajectories, there could be (1) over-burdened satellites that retain excessive amounts of data and (2) under-utilized satellites that have spare contact time to GS. In these situations, ISL can be utilized eventually download more data. For example, an over-burdened satellite A can offload its data to an under-utilized satellite B through ISL. After that, the under-utilized satellite B can deliver the received data to the GS during its contact time.

We consider a set of GSs, $K=\{k_{1},k_{2},...,k_{K}\}$, and define the two-dimensional Cartesian coordinates (i.e., xy-coordinate) of GS k∈K with altitude 0 as $p_{k}=\left(x_{k},y_{k}\right)$. We also consider a set of LEO satellites, $L=\{l_{1},l_{2},...,l_{L}\}$. We assume that all satellites have the same and unvarying altitudes *h* during an episode T={1,2,...,T}, and define the xy-coordinate of satellite l∈L at time slot t∈T as $p_{l}\left(t\right)=\left(x_{l}\left(t\right),y_{l}\left(t\right)\right)$.

For simplicity, we assume that SGLs/ISLs can be established when GSs and satellites are within a certain distance each other. Specifically, satellite *l* can deliver data to GS *k* through SGL if their Euclidean distance $d_{lk}\left(t\right)={\left|p_{l}\left(t\right)-p_{k}\right|}_{2}\leq d_{\mathrm{th}}^{\mathrm{SGL}}$, where ${\left|\cdot \right|}_{2}$ denotes L2 norm and $d_{\mathrm{th}}^{\mathrm{SGL}}$ is the maximal distance for SGL. Similarly, satellite *l* can offload data to another satellite $l^{\prime }$ through ISL when the Euclidean distance $d_{ll^{\prime }}\left(t\right)={\left|p_{l}\left(t\right)-p_{l^{\prime }}\left(t\right)\right|}_{2}\leq d_{\mathrm{th}}^{\mathrm{ISL}}$, where $d_{\mathrm{th}}^{\mathrm{ISL}}$ is the maximal distance for ISL. We also assume that satellites can transmit one data unit at each time slot, and SGL/ISL have the same data rates, as in [22].

## 3. Deep-Reinforcement-Learning-Based Cooperative Downloading Scheme

In this section, we propose a DRL-based cooperative downloading scheme. We first explain the overview of the scheme, and formulate the cooperative downloading problem as MDP. After that, we describe a discretized SAC-based training algorithm to train the optimal policy.

### 3.1. Overview

To maximize the amount of downloaded data, all of the satellites have to fully utilize their contact times with GSs. However, satellites have different amounts of data and contact times according to their trajectories. Although ISL can be utilized to overcome mismatches between their contact time and retaining data, the high mobility of LEO satellites and its impacts on the dynamic formation of ISL/SGL make it more challenging. In this regard, we formulate MDP to obtain the optimal policy, which makes LEO satellite networks take the optimal decision regarding whether to offload or download data depending on the currently observed network state information, which ultimately leads to maximizing the amounts of downloaded data.

**Figure 2:**
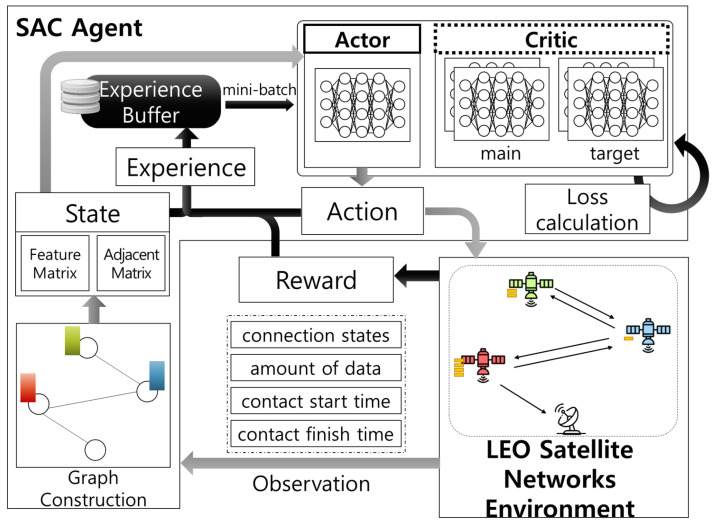
DRL-based cooperative downloading framework.

Figure 2 shows the overall DRL-based cooperative downloading framework, which is composed of LEO satellite network environments and an SAC agent with an actor network to approximate the optimal cooperative downloading policy and critic networks to evaluate the policy. At the beginning of every time slot, the SAC agent defines a state with currently observed network state information from the network environments as a graph. The SAC agent then utilizes the actor network to map the state into an action and controls the environments with the action. Its corresponding results are observed as a reward and a transition of the state (i.e., next state). The SAC agent stores all of this information (i.e., a tuple (state, action, reward, next state)) as an experience to the experience buffer. At the end of each time slot, the agent samples a mini-batch of a few experiences from the experience buffer and calculates losses to train the optimal policy. The detailed procedure is elaborated in Section 3.3.

**Figure 3:**
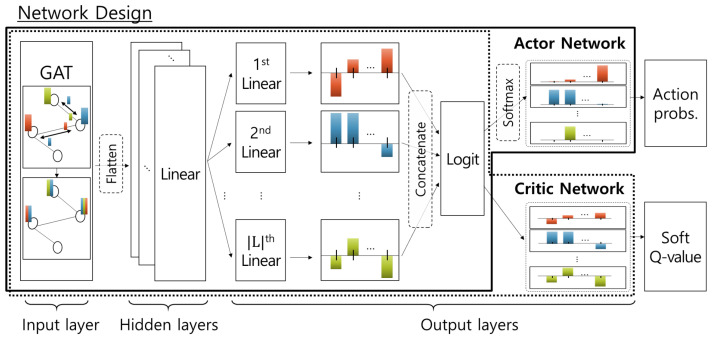
Neural network designs.

Meanwhile, the actor and critic networks follow identical neural network designs as shown in Figure 3, which consists of (1) an input layer with GAT [33], (2) hidden layers with FC layers, and (3) output layers with parallel FC layers. At the input layer, GAT, which is one of the graph convolutional networks, is adopted to efficiently process graph-oriented network states. It propagates the feature vector of each node to neighboring nodes and aggregates received vectors with an attention mechanism. After that, latent features of the graph-based states are extracted while maintaining the relationship between nodes. The following FC layers of hidden layers merge the extracted latent features obtained from each node and extract hidden features with respect to the entire graph. Lastly, in the output layer, each of |L| parallel FC layers maps the hidden features into logit for each LEO satellite. In the case of the critic network, the logit is utilized as soft-*Q* values. Meanwhile, for the actor network, it is mapped into the action probabilities by applying a softmax function.

### 3.2. MDP Formulation

To obtain the optimal cooperative downloading policy maximizing the amount of downloaded data from satellites to GS, we formulate an MDP consisting of state, action, and reward.

#### 3.2.1. State

The current state of satellite networks should represent the amount of retaining data and connection information of satellites. First of all, to effectively capture the topological relationship, a satellite network is represented as an undirected graph $G=\left(V,E\right)$, where V=L∪K denotes a set of entire nodes in the satellite network and $E=\left\{\left(v_{i},v_{j}\right)|v_{i},v_{j}\in V\right\}$ denotes a set of entire links. The time-varying feature of nodes and the shape of the graph are determined by a feature matrix $F\left(t\right)\in R^{\left|V\right|\times \left(1+2|V|\right)}$ and an adjacency matrix $A\left(t\right)\in R^{\left|V|\times |V\right|}$, respectively. Specifically, each row of the feature matrix represents a feature vector of each node. The feature vector $F_{v}\left(t\right)$ of node v∈V is defined as

(1) $$\begin{array}{c} F_{v}\left(t\right)=\left[m_{v}\left(t\right)||c_{v}^{0}\left(t\right)\left|\right|c_{v}^{1}\left(t\right)\right]\in R^{\left(1+2|V|\right)}, \end{array}$$

where ·||· denotes the concatenation symbol and $m_{v}\left(t\right)\in R$ represents the amount of retaining data. $c_{v}^{0}\left(t\right)\in R^{\left|V\right|}$ denotes the remaining time until the contact with all other nodes begins, and $c_{v}^{1}\left(t\right)\in R^{\left|V\right|}$ denotes the remaining time until the contact with all other nodes is terminated. Note that, as satellites follow their assigned orbits, it is easy to obtain such contact information of satellites [22]. The adjacency matrix A(t) is symmetric, and its element $A_{ij}\left(t\right)\in \left\{0,1\right\}$ represents whether there is a link between node $v_{i}$ and node $v_{j}$, which can be defined as

(2) $$A_{ij}\left(t\right)=\left\{\begin{array}{cc} 1, & \mathrm{if}\;v_{i},v_{j}\in L\;\mathrm{and}\;d_{ij}\left(t\right)\leq d_{\mathrm{th}}^{\mathrm{ISL}}\\ 1, & \mathrm{if}\;v_{i}\in L,v_{j}\in K,\;\mathrm{and}\;d_{ij}\left(t\right)\leq d_{\mathrm{th}}^{\mathrm{SGL}}\\ 0, & \mathrm{otherwise}. \end{array}\right.$$

Finally, the state $s_{t}$ is defined with the above-mentioned feature matrix and adjacency matrix as follows

(3) $$\begin{array}{c} s_{t}=\left\{F(t),A(t)\right\}. \end{array}$$

#### 3.2.2. Action

$a_{t}$ represents a set of actions that are performed by LEO satellites at time slot *t*, which is given by

(4) $$\begin{array}{c} a_{t}=\left\{a_{l}\left(t\right)|l\in L\right\}, \end{array}$$

where $a_{l}\left(t\right)\in V$ represents the destination of the data of satellite *l*. For example, if $a_{l}\left(t\right)=l^{\prime }\in L\setminus \left\{l\right\}$, satellite *l* attempts to transmit to another satellite $l^{\prime }$ through ISL. On the other hand, if $a_{l}\left(t\right)=k\in K$, satellite *l* attempts to download to GS *k* through SGL.

#### 3.2.3. Reward

According to the current state and the set of selected actions, the agent controls LEO satellites and receives an instantaneous reward. To obtain more rewards, we need to maximize the total amount of downloaded data during an episode. Let $n_{l}^{D}\left(t\right)$ be the amount of downloaded data of satellite l∈L at time slot *t*. We assume that each satellite can transmit one data unit at each time slot. To transmit data to GS, satellite *l* has to have sensory data and its selected action needs to indicate one of the adjacent GSs. Therefore, $n_{l}^{D}\left(t\right)$ can be expressed as

(5) $$\begin{array}{c} n_{l}^{D}\left(t\right)=\delta \left[a_{l}\left(t\right)=k,A_{lk}\left(t\right)=1,\;\mathrm{and}\;m_{l}\left(t\right)\geq 1\right],k\in K, \end{array}$$

where $\delta \left[\cdot \right]$ denotes the delta function that returns 1 if the conditions in brackets are true; otherwise, it returns 0.

Meanwhile, we need to avoid meaningless data exchange between satellites, which also affects the reward. Let $n_{l}^{O}\left(t\right)$ be the amount of offloaded data of satellite l∈L at time slot *t*. Satellite *l* can offload data only when it has data and its selected action refers to one of the adjacent satellites. Therefore, $n_{l}^{O}\left(t\right)$ can be expressed as

(6) $$\begin{array}{cc} n_{l}^{O}\left(t\right)=\delta [a_{l}\left(t\right)=l^{\prime },A_{ll^{\prime }}\left(t\right)=1,\;\mathrm{and}\; & m_{l}\left(t\right)\geq 1],l^{\prime }\in L\setminus \left\{l\right\}. \end{array}$$

To summarize, the instantaneous reward $r_{t}:=r\left(s_{t},a_{t}\right)$ at time slot *t* can be defined as

(7) $$\begin{array}{c} r_{t}=\sum_{l\in L}\left\{n_{l}^{D}\left(t\right)+\lambda \cdot n_{l}^{O}\left(t\right)\right\}, \end{array}$$

where λ≤0 denotes the weight of the offloading data.

### 3.3. Discretized SAC-Based Learning Algorithm

To train the optimal cooperative downloading policy to maximize the amount of downloaded data, we adopt the SAC-based algorithm [34]. The SAC algorithm includes an entropy term in the training objective function that evaluates the policy and trains it to maximize not only the accumulated reward but also the entropy.

We first define an actor network $\pi_{\phi }\left(\cdot \right)$ with parameter ϕ, main critic networks $Q_{\theta_{i}}$ with parameters $\theta_{i}$, and target critic networks $Q_{{\hat{\theta }}_{i}}$ with parameters ${\hat{\theta }}_{i}$ for i∈{1,2}. The detailed procedure of the SAC-based algorithm is shown in Algorithm 1. First, the algorithm initializes the experience experience buffer *D*, the weight α, and the parameters (i.e., ϕ, $\theta_{i}$, and ${\hat{\theta }}_{i}$ for i∈{1,2}) (see lines 1–5 in Algorithm 1). For each episode, the algorithm observes the initial state $s_{t}$ (see line 7 in Algorithm 1). In addition, in each time slot *t*, the algorithm generates an action $a_{t}$ by using the actor network, i.e., $a_{t}=\pi_{\phi }\left(s_{t}\right)$ (see line 9 in Algorithm 1), and observes the next state $s_{t+1}$ and the reward $r_{t}$ after executing the action $a_{t}$ (see lines 9–11 in Algorithm 1). As a result, the algorithm stores the experience $\left(s_{t},a_{t},r_{t},s_{t+1}\right)$ at the experience buffer *D* (see line 12 in Algorithm 1).
**Algorithm 1** Discretized soft-actor-critic algorithm.1:Initialize the experience buffer *D*2:Initialize the weight α with 13:Initialize the actor network $\pi_{\phi }$ with random parameter ϕ4:Initialize main critic networks $Q_{\theta_{i}}$ with random parameters $\theta_{i}$ for $i\in \left\{1,2\right\}$5:Initialize target critic networks $Q_{{\hat{\theta }}_{i}}$ with parameters ${\hat{\theta }}_{i}$ as main critic networks $Q_{\theta_{i}}$6:**for** each training episode **do**7:   Observe initial state $s_{t}$8:   **for** each step t=1,2,...,T **do**9:     Generate the action $a_{t}=\pi_{\phi }\left(s_{t}\right)$10:     Execute the action $a_{t}$11:     Observe the next state $s_{t+1}$ and the reward $r_{t}$12:     Store the experience $\left(s_{t},a_{t},r_{t},s_{t+1}\right)$ at the experience buffer *D*13:     Sample a mini-batch $\bar{D}$ of a few experiences from the buffer *D*14:     Calculate the target state value $V_{\hat{\theta }}\left(s^{\prime }\right)$ based on Equation (9)15:     Update the main critic network $Q_{\theta_{i}}$ based on the gradient $\nabla_{\theta_{i}}J_{Q}\left(\theta_{i}\right)$ in Equation (10)16:     Update the actor network $\pi_{\phi }$ based on the gradient $\nabla_{\phi }J_{\pi }\left(\phi \right)$ in Equation (12)17:     Update the weight α based on the gradient $\nabla_{\alpha }J\left(\alpha \right)$ in Equation (13)18:     For every *B* steps, use soft update for the target critic networks based on Equation (14)19:   **end for**20:**end for**

Now the algorithm enters into the parameter update phase. To this end, the algorithm randomly samples a mini-batch $\bar{D}$ of a few experiences from the buffer *D* (see line 13 in Algorithm 1). Based on these sampled experiences, the algorithm calculates the target state value $V_{\hat{\theta }}\left(s^{\prime }\right)$ as

(8) $$\begin{array}{c} V_{\hat{\theta }}\left(s^{\prime }\right)=E_{{\tilde{a}}^{\prime }∼\pi_{\phi }\left(s^{\prime }\right)}\left[Q_{\hat{\theta }}^{min}\left(s^{\prime },{\tilde{a}}^{\prime }\right)-\alpha \log\left(\pi_{\phi }\left(s^{\prime }\right)\right)\right], \end{array}$$

where $s^{\prime }$ denotes the next state of a tuple $\left(s,a,r,s^{\prime }\right)\in \bar{D}$, which is one of the mini-batch samples and ${\tilde{a}}^{\prime }$ denotes an action obtained from $s^{\prime }$ and the actor network $\pi_{\phi }\left(\cdot \right)$. In addition, $Q_{\hat{\theta }}^{min}\left(s^{\prime },\tilde{a}\right)$ represents the minimum value between $Q_{{\hat{\theta }}_{1}}\left(s^{\prime },\tilde{a}\right)$ and $Q_{{\hat{\theta }}_{2}}\left(s^{\prime },\tilde{a}\right)$. Note that the generated actions by the cooperative downloading policy should be the indices for GSs or adjacent LEOs receiving the data. That is, the policy should be defined over a discrete action space, and, therefore, we adopt an SAC parameter update algorithm considering discrete action spaces [35]. Specifically, the output layers of neural networks are organized with |L| FC layers in parallel so that each layer corresponds to each satellite, and the output size of each layer is set as |V| so that each output corresponds to the discretized action space. These network designs allow the actor and critic networks to map their states into the action probabilities and Q-values, respectively. Consequently, the expectation operation in (8) can be replaced with the direct output of the actor network without deriving any action probability density function. That is, the target state value in the discretized action spaces can be computed as (see line 14 in Algorithm 1)

(9) $$\begin{array}{c} V_{\hat{\theta }}\left(s^{\prime }\right)=\pi_{\phi }{\left(s^{\prime }\right)}^{⊤}\left(Q_{\hat{\theta }}^{min}\left(s^{\prime },\cdot \right)-\alpha \log\left(\pi_{\phi }\left(s^{\prime }\right)\right)\right). \end{array}$$

Note that the transformed critic networks $Q_{\hat{\theta }}^{min}\left(s^{\prime },\cdot \right)$ do not require the action $a^{\prime }$ as an input. This is because the critic networks generates soft *Q*-values over selectable actions directly. Based on the target state value, the algorithm updates the main critic networks (see line 15 in Algorithm 1) with a gradient

(10) $$\begin{array}{cc} \nabla_{\theta_{i}}J_{Q}\left(\theta_{i}\right)=\nabla_{\theta_{i}}E & {}_{\left(s,a,r,s^{\prime }\right)∼\bar{D}}\left[\frac{1}{2}{\left(Q_{\theta_{i}}\left(s,a\right)-\left(r+\psi E_{s^{\prime }}\left[V_{\hat{\theta }}\left(s^{\prime }\right)\right]\right)\right)}^{2}\right]. \end{array}$$

Furthermore, the algorithm updates the actor network with a gradient

(11) $$\begin{array}{c} \begin{array}{c} \nabla_{\phi }J_{\pi }\left(\phi \right)\approx \nabla_{\phi }E_{s∼\bar{D}}\left[E_{\tilde{a}∼\pi_{\phi }\left(s\right)}\left[\alpha \log\left(\pi_{\phi }\left(s\right)\right)-Q_{\theta }^{min}\left(s,\tilde{a}\right)\right]\right]. \end{array} \end{array}$$

By the same discretization, the inner expectation of (11) can also be calculated directly, and, thus, (11) can be rearranged as

(12) $$\begin{array}{c} \begin{array}{c} \nabla_{\phi }J_{\pi }\left(\phi \right)\approx \nabla_{\phi }E_{s∼\bar{D}}\left[\pi_{\phi }{\left(s\right)}^{⊤}\left(\alpha \log\left(\pi_{\phi }\left(s\right)\right)-Q_{\theta }\left(s,\cdot \right)\right)\right]. \end{array} \end{array}$$

Then, the algorithm updates the actor network accordingly (see line 16 in Algorithm 1).

Additionally, the weight α is automatically adjusted as the training progresses (see line 17 in Algorithm 1), with a gradient

(13) $$\begin{array}{c} \nabla_{\alpha }J\left(\alpha \right)=\nabla_{\alpha }\pi_{\phi }{\left(s\right)}^{⊤}\left(-\alpha \left(\log\left(\pi_{\phi }\left(s\right)\right)+\hat{H}\right)\right), \end{array}$$

where $\hat{H}$ denotes the target entropy.

Finally, the algorithm updates the target critic networks with the main critic networks by using an exponentially moving average (i.e., soft update) for every *B* steps (see line 18 in Algorithm 1) as

(14) $$\begin{array}{c} {\hat{\theta }}_{i}=\tau \cdot \theta_{i}+\left(1-\tau \right)\cdot {\hat{\theta }}_{i},i\in \left\{0,1\right\}, \end{array}$$

where τ denotes a coefficient for the soft update.

## 4. Performance Evaluation

For performance evaluation, we compare the proposed scheme, DRL-CD, with the following schemes: (1) RANDOM, where each LEO satellite makes offloading decisions randomly until it begins to contact GS, and (2) No-COOP, which does not support any ISL offloading.

**Figure 4:**
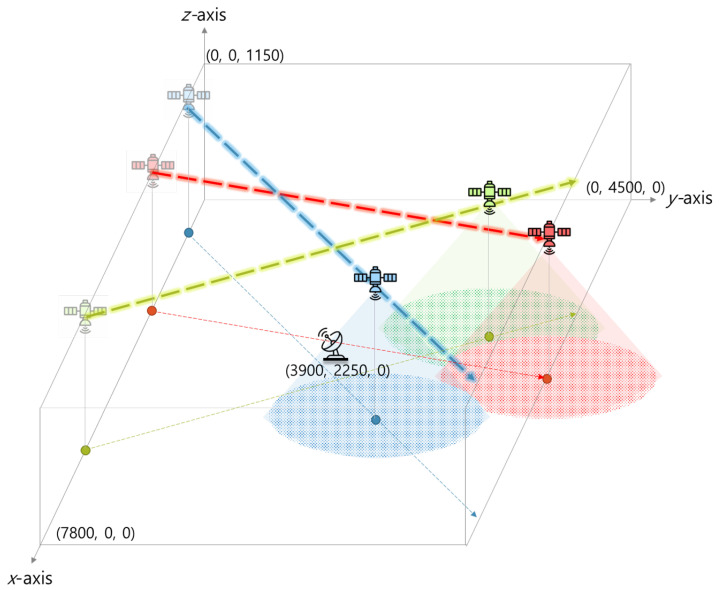
Illustration of a simulation environment.

We assume that the altitude of satellite is 1150 km and consider a target area of 7800 × 4500 ×1150 (${\mathrm{km}}^{3}$) in which one GS is deployed at the center of the area as shown in Figure 4. At the beginning of each episode, we generate random orbits that intersect around GS to prevent satellites from passing through the target area without any contact. The elevation angle of GS is set to 35${}^{\circ }$, resulting in the maximal SGL distance (denoted by $d_{\mathrm{th}}^{\mathrm{SGL}}$) of 1230 km [36]. In addition, $d_{\mathrm{th}}^{\mathrm{ISL}}$ is set to 1865 km, which can be computed based on the maximum distance between two satellites that move along adjacent planes within one constellation. When the inclination of the constellation is 53${}^{\circ }$ and the difference in the right ascension of the ascending nodes of the two satellites is 8${}^{\circ }$, the maximum distance is 1865 km [37]. The bandwidths of SGL and ISL are set to 33 MHz in the C-band of 5.1–5.2 GHz and 16.5 MHz in the L-band of 6.2–6.5 GHz, respectively [22].

We composed the input layer, the hidden layers, and the output layers of the DNN with 1 GAT layer, 4 FC layers, and |L| paralleled FC layers, respectively. Other simulation parameters are summarized in Table 1.

The optimal cooperative downloading policy allows data to be distributed to satellites to fully utilize satellites’ contact time and maximize the amount of downloaded data. To quantify the distribution of data that satellites initially retain, the G-fairness index is defined as

(15) $$\begin{array}{c} I\left(m\right)=\prod_{l\in L}\sin\left(\frac{\pi \cdot m_{l}}{2\cdot \max(m)}\right), \end{array}$$

where $m=\{m_{l}|l\in L\}$ denotes a set of initial amounts of data that satellites retain [38]. If each LEO satellite has comparable amounts of data (i.e., the total amount of data is distributed in a balanced manner over the satellites), I(m) is close to 1; otherwise, it is close to 0. At the beginning of every episode, the total amount of initial data is set as the sum of the maximally downloadable data of each trajectory and distributed to satellites according to I(m).

### 4.1. Effect of Initial Data Distribution

**Figure 5:**
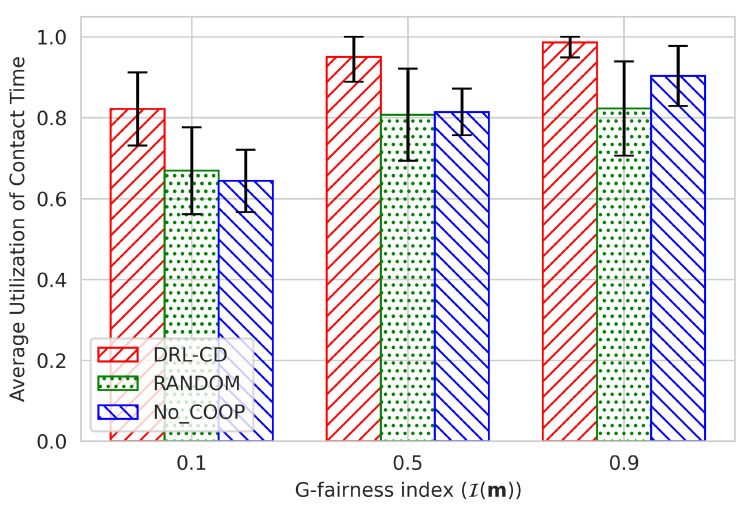
G-fairness index vs. average contact-time utilization.

Figure 5 shows the effect of the G-fairness index on the average utilization of the contact time with GS. We consider three LEO satellites and evaluate their performance with the average results when the orbits of 100 episodes are randomly generated. From Figure 5, it can be found that the contact time utilization decreases as the G-fairness index decreases (i.e., the initial data distribution is biased). This is because biased data distributions require more time to offload data from over-burdened satellites to under-utilized ones. Moreover, it is difficult to guarantee sufficient offloading time in dynamic satellite networks.

It can be also seen that DRL-CD always exhibits higher contact-time utilization than other schemes. Specifically, in the case of balanced data distributions (i.e., I(m)=0.9), DRL-CD utilizes the contact time 16.4% and 8.2% more efficiently than RANDOM and No-COOP, respectively. Meanwhile, for biased data distributions (i.e., I(m)=0.1), DRL-CD shows improved contact-time utilization by 15.2% and 17.8% compared to RANDOM and No-COOP, respectively. This is because DRL-CD can successfully train the cooperative downloading policy that makes all satellites offload appropriate amounts of data and efficiently utilize the given contact time regardless of the initial data distribution.

### 4.2. Effect of Number of Satellites

**Figure 6:**
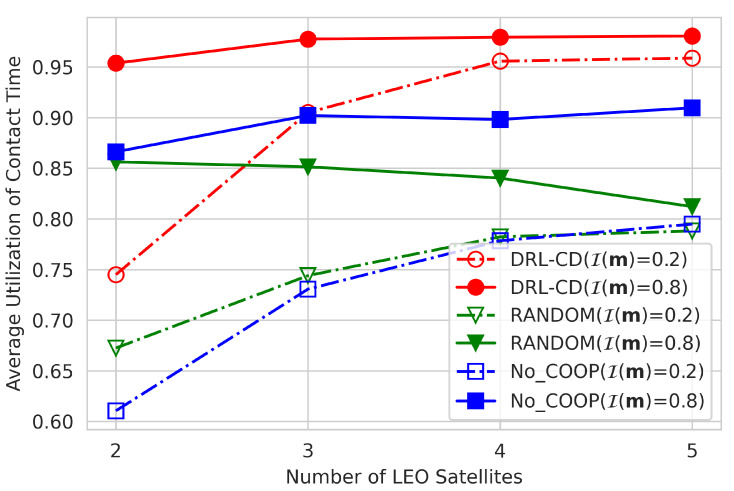
Number of satellites vs. average contact-time utilization.

Figure 6 shows the effect of the number of LEO satellites on the average contact-time utilization when 100 episodes are randomly generated and the G-fairness index is set to 0.2 or 0.8. From Figure 6, it can be found that the average contact-time utilization generally increases as the number of satellites increases. For example, in case of highly biased data distributions (i.e., I(m)=0.2), the contact-time utilizations of DRL-CD, RANDOM, and No-COOP increase by 21.4%, 11.5%, and 18.4%, respectively, when the number of LEO satellites is changed from 2 to 5. This is because the amount of downloaded data in the cooperative downloading highly depends on the contact time not only with GS but also with other satellites. The higher the number of satellites, the higher the probability of providing opportunities for ISL offloading, which allows more data to be offloaded from over-burdened satellites to under-utilized ones. Consequently, DRL-CD becomes more effective by deploying more LEO satellites, which is promising since more and more LEO satellites will be launched in the future.

## 5. Conclusions

In this paper, we proposed DRL-based cooperative downloading for LEO satellite networks. We first modeled the satellite networks as a graph to capture the generalized network states and formulated the MDP problem for cooperative downloading. To solve the formulated problem, we adopted the SAC-based training algorithm for discrete action space and design neural networks. Evaluation results show that the proposed DRL-based cooperative downloading scheme can guarantee higher utilization of satellites’ contact time by up to 17.8% compared with other schemes. We adopted a centralized reinforcement learning assuming all the network state information (i.e., channel state information of all the links and satellites states) can be observed at the centralized agent. However, it is challenging to collect network-wide observations, as LEO satellites have high mobility and follow their own orbits. To address this issue, in our future work, we will extend our scheme to include multi-agent reinforcement learning in which each satellite operates as an agent, trains the optimal policy with few or minimal data exchanges, and makes decisions only with locally observable network state information. In addition, IRS is a promising technology to improve routing, link performances, and energy efficiency in multi-layer satellite networks. Thus, we will consider IRS-assisted download services in our future work.

## Figures and Tables

**Table 1:** Parameters.

Parameter	Value
Neurons of each hidden layer	512
Neurons of each output layer	number of satellites + number of GSs
Batch size	128
Replay buffer size	1,000,000
Learning rate	3 × 10${}^{-4}$
Discount rate	0.99
Optimizer	Adam
Target entropy	0.98∗log(numberofsatellites)
Weight for offloading (λ)	−0.3
Soft update cycle (*B*)	2
Coefficient for soft update (τ)	5 × 10${}^{-3}$

## Data Availability

Not applicable.
